# The effects of hemorrhagic parenchymal infarction on the establishment of sensori-motor structural and functional connectivity in early infancy

**DOI:** 10.1007/s00234-014-1412-5

**Published:** 2014-08-14

**Authors:** T. Arichi, S. J. Counsell, A. G. Allievi, A. T. Chew, M. Martinez-Biarge, V. Mondi, N. Tusor, N. Merchant, E. Burdet, F. M. Cowan, A. D. Edwards

**Affiliations:** 1 Department of Perinatal Imaging & Health, Division of Imaging Sciences & Biomedical Engineering, Kings College London, St Thomas’ Hospital, 1st floor North Wing, Westminster Bridge Road, London, SE1 7EH UK; 2 Department of Bioengineering, Imperial College London, London, UK; 3 Department of Paediatrics, Imperial College Healthcare NHS Trust, London, UK

**Keywords:** Neonatal, Focal brain lesion, fMRI, Diffusion MRI, Connectivity

## Abstract

**Introduction:**

The objective of the study was to characterize alterations of structural and functional connectivity within the developing sensori-motor system in infants with focal perinatal brain injury and at high risk of cerebral palsy.

**Methods:**

Functional magnetic resonance imaging (fMRI) and diffusion tensor imaging (DTI) data were used to study the developing functional and structural connectivity framework in six infants born prematurely at term equivalent age. This was first characterised in three infants without focal pathology, which was then compared to that derived from three infants with unilateral haemorrhagic parenchymal infarction and a subsequent focal periventricular white matter lesion who developed later haemiparesis.

**Results:**

Functional responses to passive hand movement were in the contralateral perirolandic cortex, regardless of focal pathology. In infants with unilateral periventricular injury, afferent thalamo-cortical tracts appeared to have developed compensatory trajectories which circumvented areas of damage. In contrast, efferent corticospinal tracts showed marked asymmetry at term equivalent age following focal brain injury. Sensori-motor network analysis suggested that inter-hemispheric functional connectivity is largely preserved despite pathology and that impairment may be associated with adverse neurodevelopmental outcome.

**Conclusion:**

Following focal perinatal brain injury, altered structural and functional connectivity is already present and can be characterized with MRI at term equivalent age. The results of this small case series suggest that these techniques may provide valuable new information about prognosis and the pathophysiology underlying cerebral palsy.

**Electronic supplementary material:**

The online version of this article (doi:10.1007/s00234-014-1412-5) contains supplementary material, which is available to authorized users.

In the third trimester of gestation, the brain’s connectivity framework is established through a complex but highly programmed sequence of maturation, mediated through key transient developmental structures such as the subcortical subplate [1]. The importance of this period for life-long brain structure and function is emphasized by marked increases in the prevalence of neurodevelopmental impairment in survivors of preterm birth, in comparison to their peers born at full term [2]. Recent advances in imaging have now made it possible to non-invasively characterize the large-scale architecture of the brain’s emerging pattern of functional and structural connectivity using blood oxygen level dependent (BOLD) functional magnetic resonance imaging (fMRI) and diffusion-weighted MRI [3]. Significantly, while such connectivity measures have been found to be altered in relation to clinical outcome in the mature brain, the specific effects of focal pathology on the establishment of intrinsic connectivity within the developing brain during early infancy have not previously been explored [4, 5].

Preterm infants are at risk of intra-ventricular haemorrhage (IVH) and periventricular haemorrhagic parenchymal infarction (HPI) [6]. The developing sensori-motor system is particularly vulnerable as its pathways course through the commonest site of injury, with unilateral HPI typically resulting in cerebral palsy and a contralesional haemiparesis [6]. However, there have also been a number of case reports describing altered patterns of functional activity in later childhood following perinatal brain injury, which may reflect the newborn brain’s increased capacity for neural plasticity and reorganization [7, 8]. In this study, we aimed to characterize the specific effects of HPI on the developing sensori-motor system of infants with later cerebral palsy by precisely localizing functional responses to a passive motor task with fMRI, by delineating the afferent thalamo-cortical and efferent corticospinal tracts (CSTs) with diffusion tensor imaging (DTI) probabilistic tractography and by exploring intrinsic functional connectivity within a putative sensori-motor network. These techniques were first applied to provide a characterization of the sensori-motor connectivity framework in the absence of focal pathology in three ‘control’ prematurely born infants with normal motor outcome. They were then used to study three ‘case’ infants with unilateral HPI and a subsequent periventricular white matter lesion, who later developed contralateral spastic cerebral palsy. We hypothesized that in comparison to infants without focal brain injury, topological and quantitative differences in functional and structural connectivity would already be present at term equivalent age (TEA) in infants with HPI, which would be consolidated at 1 year of age.

## Method

The work was approved by the NHS Research Ethics Committee, and written parental consent was obtained prior to all data acquisition. Data were collected at the Queen Charlotte and Chelsea Hospital, London, UK between 2009 and 2012.

### Participants

The study population consisted of six prematurely born infants: three infants known not to have focal brain pathology (controls) (selected from a previously described cohort [9]) and three infants diagnosed on cranial ultrasound examination with unilateral HPI (cases) and a consequent focal periventricular white matter lesion. All six infants were studied using MRI at TEA, and case infants were additionally studied at 1 year of corrected age (see Table 1). All infants were seen at 1-year corrected age for a neurodevelopmental assessment using the Griffiths Mental Developmental Scale (revised) (GMDS-R) and an age-appropriate standardized neurological examination [10–12].

**Table 1:** Clinical characteristics and neurodevelopmental outcome of the study population

Subject number	Gestation at birth (weeks + days)	Birth weight (g)	Birth occipito-frontal head circumference (cm)	Clinical interpretation of structural MR images at TEA	Clinical history in the neonatal period	Neurological outcome at 1 year	Neurodevelopmental outcome (developmental quotient score from GMDS-R)
Control 1	26 + 4	955	23	Appropriate for post-menstrual age, no focal brain lesion	Maternal sepsis Twin 1 of dichorionic diamniotic twins (DCDA) Conservatively treated patent ductus arteriosus (PDA) Chronic lung disease (CLD)	No focal asymmetry or evidence of major motor problem	102.7
Control 2	29 + 1	970	25.8	Appropriate for post-menstrual age, no focal brain lesion	Maternal pre-eclampsia Thrombocytopenia Coagulase-negative staphylococcus sepsis (CNSS)	No focal asymmetry or evidence of major motor problem	104
Control 3	25 + 5	815	23	Appropriate for post-menstrual age, no focal brain lesion	Spontaneous preterm labour CNSS Pulmonary haemorrhage, CLD Grade 2 retinopathy of prematurity (ROP) Grade 1 intra-ventricular haemorrhage (IVH)	No focal asymmetry or evidence of major motor problem	102.5
Focal lesion case 1	26 + 2	980	24.5	Right-sided haemorrhagic parenchymal infarction with involvement of the right thalamus and PLIC. Dilatation of the ventricles (right > left and posterior more than anterior) and white matter punctate lesions bilaterally.	Maternal sepsis Conservatively treated PDA Right grade 4 IVH	Left haemiparesis, lower limb bilateral spastic cerebral palsy, marked truncal hypotonia (could not stand or sit unsupported)	54.7
Focal lesion case 2	26 + 1	1,030	23.5	Left-sided haemorrhagic parenchymal infarction with left thalamic atrophy and decreased myelination in the left PLIC. Bilateral periventricular punctate lesions and diffuse high signal on T2 images in the white matter.	Maternal sepsis CNSS PDA (treated with ligation) CLD Grade 4 ROP Left grade 4 IVH	Right haemiparesis, mild truncal hypotonia (could stand supported)	76.4
Focal lesion case 3	27 + 6	1,060	25.1	Right-sided haemorrhagic parenchymal infarction with ventricular dilatation, right thalamic atrophy and lack of myelination in the right PLIC and brainstem.	Twin 2 of DCDA twins Congenital *E. coli* septicaemia Right grade 4 IVH	Left haemiparesis, mild truncal hypotonia (could stand and walk supported)	106.3

### MR image acquisition

Infants were clinically assessed by a paediatrician, who was in attendance throughout the MRI scan. All infants were given chloral hydrate sedation (30–50 mg/kg at TEA; 50–80 mg/kg at 1-year corrected age) 20 min prior to image acquisition. During scanning, temperature, oxygen saturations and heart rate were monitored, hearing protection was applied (dental putty and adhesive ear muffs (MiniMuffs, Natus Medical Inc., San Carlos, CA, USA), and the head was immobilized with a vacuum-evacuated polystyrene-bead-filled pillow [13].

MRI data were collected with a 3-T Philips Achieva MRI system (Best, the Netherlands), with an eight-channel phased-array head coil. High-resolution conventional images were acquired using a turbo spin echo (TSE) T2-weighted sequence and a 3D magnetization-prepared rapid acquisition gradient echo (MP-RAGE) T1-weighted sequence and reviewed by a perinatal neuroradiologist [13]. fMRI data were collected with a single-shot gradient echo echo-planar imaging (EPI) sequence lasting 6 min and 34 s (parameters: in-plane resolution 2.5 mm * 2.5 mm; 22 slices; slice thickness 3.25 mm; repetition time (TR) 1,500 ms; echo time (TE) 45 ms; flip angle (FA) 90°; total 256 volumes). DTI data were collected in 32 non-collinear directions with a *b* value of 750 s/mm^2^ using a spin echo EPI sequence (parameters: in-plane resolution 1.75 mm * 1.75 mm; 49 slices; slice thickness 2 mm; TR 9,000 ms; TE 49 ms). Passive sensori-motor stimulation for the fMRI experiment was performed using a simple block paradigm (24 s of passive movement alternating with 24 s of rest) and a fully automated hand interface in the form of a custom-made inflatable balloon which was placed in the palm contralateral to the known periventricular lesion in case infants (or right hand in controls) [9]. Inflation of the balloon resulted in passive finger extension (and deflation in finger flexion), and the timing of stimulation was fully synchronized with fMRI data acquisition [9].

### Image analysis

Image analysis was performed using tools implemented in the FMRIB Software Library (FSL, Oxford, UK) (http://fsl.fmrib.ox.ac.uk/fsl/fslwiki/) [14]. Standard preprocessing steps as implemented in FEAT v5.98 were performed on the fMRI data including motion correction, spatial smoothing (5-mm FWHM), highpass temporal filtering (cut-off 50 s) and data denoising using MELODIC (Model-free FMRI analysis using probabilistic independent component analysis (PICA, v3.0)) [14]. Time-series statistical analysis was performed using the general linear model (GLM), with the measured data represented by a convolution of the experimental design and an age-appropriate basis set generated using FLOBS (FMRIB’s linear optimal basis sets) [14, 15]. Calculated parameter estimates were then converted to a *z*-statistical score image (threshold 2.3) with a corrected cluster significance level of *p* < 0.05.

The afferent thalamo-cortical and efferent CSTs were delineated by probabilistic tractography using tools implemented in FSL’s Diffusion Toolbox (FDT) [14]. Image volumes representing gradient directions corrupted by motion artefact during acquisition were excluded from the total data set [16]. Standard preprocessing steps were applied (non-brain tissue removal, eddy current correction), and fractional anisotropy (FA), radial diffusivity (RD), and axial diffusivity (AD) were calculated by fitting the data to the diffusion tensor model [17]. Voxelwise diffusion parameter distributions were then calculated using Bayesian Estimation of Diffusion Parameters Obtained using Sampling Techniques, X stands for modelling crossing fibres (BEDPOSTX) [14]. The thalamo-cortical tracts were then identified using a manually delineated seed in the thalamus ipsilateral to a waypoint mask derived from the top quartile of *z* scores within the subject-specific fMRI activation cluster in the perirolandic region. This process was repeated for both hemispheres in infants with bilateral patterns of functional activity and for functional clusters in the insula where relevant. The CSTs were delineated separately using a set of anatomically defined regions (the cerebral peduncle, posterior limb of the internal capsule (PLIC) and perirolandic cortex) [18]. The identified connectivity distributions were then normalized by the number of samples passing from the seed mask through the distal waypoint areas and were thresholded at 1 % [17]. For each of the identified CSTs, volume and diffusion metrics (FA, RD and AD) were determined and an asymmetry index (AI) for these values calculated using the formula [2 * (mean diffusion metric contralesional tract − mean diffusion metric ipsilesional tract)/(mean diffusion metric contralesional tract + mean diffusion metric ipsilesional tract)] [18]. For control infants, this formula was adapted to [2 * (mean left tract − mean right tract)/(mean left tract + mean right tract)] [18].

### Functional connectivity analysis

Intrinsic functional connectivity was studied within a putative sensori-motor network consisting of nine anatomically defined 6-mm radius sphere masks. These were placed in the bilateral perirolandic areas (encompassing the primary motor (M1) and somatosensory (S1) cortices), supplementary motor area (SMA) (a single mask that contained both left and right sides), bilateral insular areas (including the overlying opercular cortex and secondary somatosensory cortex S2), bilateral basal ganglia and bilateral thalami (see Supplementary Figure) [19]. To remove the effects of stimulus-induced signal change, only the residuals from the GLM analysis were used for this analysis, as it has been previously described to give a good representation of the underlying resting state signal [20]. Further band-pass temporal filtering was not performed in view of the risk of producing edge artefacts. The mean eigen-time series was extracted from each mask, and pairwise Pearson’s partial correlation coefficients calculated using the Statistics Toolbox of MATLAB (2012a, the MathWorks, Natick, MA, USA). The six estimated head motion parameters (translations and rotations) and the cerebrospinal fluid (CSF) time series were included as confound regressors. A connectivity distribution map was then prepared using Cytoscape v3.0 (http://www.cytoscape.org/ ) using only the significant connections as identified by a false discovery rate correction (FDR) of *p* < 0.05 [21].

## Results

### Conventional MR imaging and neurodevelopmental outcome

Control infants had no focal brain lesions on conventional MR images, had no evidence of asymmetrical neurological signs and had a neurodevelopmental outcome within the normal range at 1-year corrected age (Table 1). All three cases at TEA and 1-year corrected age had a large periventricular white matter lesion at the site of the HPI, with suboptimal myelination in the PLIC and decreased thalamic volume ipsilateral to the cyst (in comparison with the non-lesional side), and the appearances were therefore highly predictive of later unilateral spastic cerebral palsy [22]. Although all case infants had a clear area of unilateral periventricular white matter injury (as a result of the HPI), there were also differing degrees of ventriculomegaly and global white matter abnormality, with case 1 the most severely affected with appearances consistent with periventricular leukomalacia (PVL). At 1-year corrected age, all cases had asymmetrical neurological signs consistent with unilateral spastic cerebral palsy. This was most severe in case 1, who also had marked global neurodevelopmental delay (overall developmental quotient (DQ) score, 54.7). In contrast, while case 3 also had asymmetrical neurological signs, neurodevelopmental assessment was within the normal range (DQ score, 106.3).

### Functional imaging

**Fig. 1:**
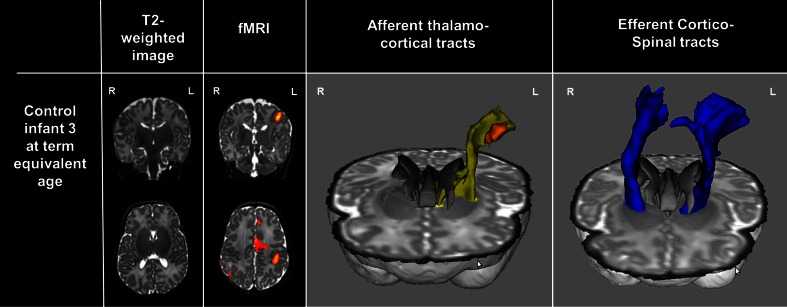
Functional activation and probabilistic tractography in a control preterm infant without focal brain injury and imaged at term equivalent age. Following passive sensori-motor stimulation of the right hand, clusters of functional activity were identified in the contralateral (*left*) perirolandic region and supplementary motor area (*z*-score threshold 2.3). The afferent thalamo-cortical tract (*yellow*) was identified using probabilistic tractography and the fMRI cluster (*orange*) as a target mask. Symmetrical efferent corticospinal tracts (*blue*) were identified using anatomical regions of interest

**Fig. 2:**
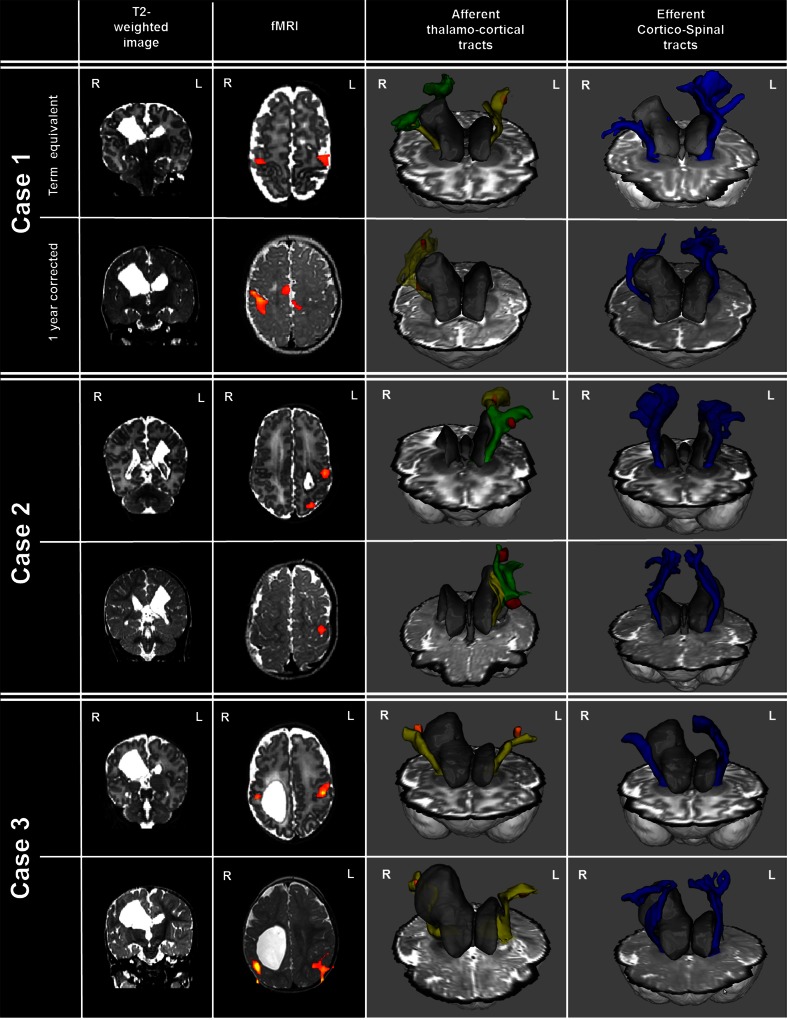
Functional activation and probabilistic tractography in three cases with focal periventricular brain injury, studied at term equivalent and 1-year corrected age. A unilateral periventricular white matter lesion can be seen arising from the lateral ventricle at the site of the previous haemorrhagic infarction on the *right* (*cases 1* and *3*) and *left* (*case 2*) sides. Following passive sensori-motor stimulation of the contralesional hand, clusters of functional activity were identified in all infants at both time-points in the ipsilesional perirolandic region (*z*-score threshold 2.3). The afferent thalamo-cortical tracts (*yellow* and *green*) developed altered trajectories which circumvented the periventricular white matter lesion to meet the identified fMRI clusters (*orange*/*red*). Efferent corticospinal tracts (*blue*) showed marked asymmetry, with a decreased volume in the lesional hemisphere evident at both time-points

In all infants at TEA, positive BOLD functional clusters were identified in the perirolandic region contralateral to the side of passive sensori-motor stimulation (Figs. 1 and 2). In control infants, responses were localized to the ‘hand’ area of the contralateral perirolandic cortex with additional clusters in the SMA and were bilateral in one infant. In case infants, responses were seen bilaterally at TEA in cases 1 and 3 but only in the lesioned hemisphere in case 2. Functional clusters were also displaced within the perirolandic cortex posteriorly (case 2) or inferiorly and laterally (case 3). Activity was not identified in the SMA in case infants, although additional clusters were seen inferiorly in the insula in case 1.

At 1-year corrected age, functional responses remained in the lesioned hemisphere in case infants (Fig. 2). Functional activity in case 1 was seen exclusively in the perirolandic region of the lesional hemisphere (having previously had bilateral S1 responses) with additional areas in the SMA, insula and parietal operculum. Cases 2 and 3 retained a similar pattern to that seen at TEA, with unilateral and bilateral responses, respectively.

### DTI tractography of the thalamo-cortical tracts

At TEA, afferent thalamo-cortical tracts could be delineated in all six infants from the thalamus running supero-laterally to the fMRI cluster in the perirolandic cortex contralateral to the side of stimulation (Figs. 1 and 2). In cases 2 and 3, the tracts had developed an altered trajectory which circumvented the area of focal periventricular white matter injury through additional curvature at both TEA and 1 year of age. In case 1 at TEA, it was not possible to delineate a tract running directly from the thalamus to the perirolandic region, although this connection was found to be possible through an indirect route of two tracts, the first of which connected the thalamus to the insula area (using the identified functional cluster in this region as a target for the tractography) and the second then connecting the insula superiorly to the area of functional activity in the perirolandic region. A single thalamo-cortical pathway could then be delineated at 1 year of age running through the insula (and passing through the functional cluster still present in the area), suggesting possible consolidation of the two pathways into a single tract.

### DTI tractography of the corticospinal tracts

**Fig. 3:**
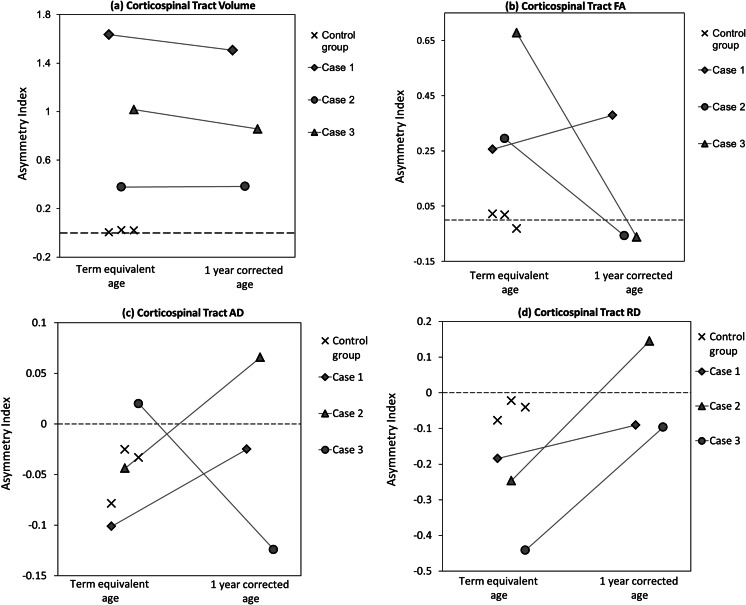
Corticospinal tract volume and microstructural integrity. **a** In comparison to control infants (*crosses*), corticospinal volume in case infants was clearly asymmetrical at both term equivalent and 1-year corrected age. **b**, **d** Markers of microstructural integrity (fractional anisotropy (*FA*) and radial diffusivity (*RD*)) were also asymmetric at term equivalent age in cases, although this was not sustained at 1 year of age. **c** There was no difference in asymmetry in axial diffusivity (*AD*) at either time-point

In each of three control infants at TEA, there was no significant asymmetry between the left and right CSTs in either volume or diffusion metrics (Fig. 3). Bilateral CSTs were also identified in all three case infants at both TEA and 1 year of age, although with marked asymmetry in volume due to a reduction in the lesional hemisphere in comparison to the intact side (Figs. 2 and 3). At TEA, marked asymmetry was also seen in FA (reduced in the lesional hemisphere) and RD (increased in the lesional hemisphere) suggesting reduced microstructural integrity (Fig. 3). Despite their clinical outcome, consistent asymmetry between the CSTs in any of the diffusion metrics was not sustained at 1 year.

### Functional connectivity of the sensori-motor network

**Fig. 4:**
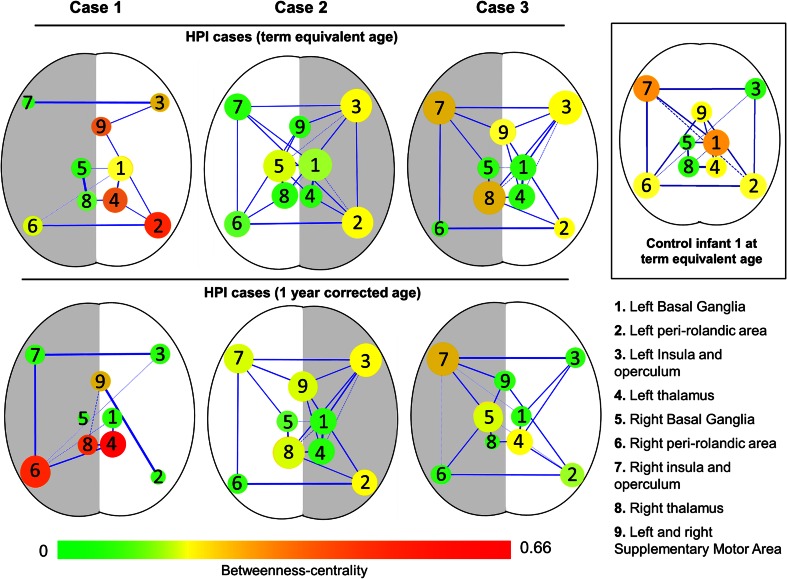
Functional connectivity in the sensori-motor network. In an example control infant (*inset box*), a clear pattern of functional connectivity can be seen between one brain region and its homotopic counterpart in the opposite hemisphere. In case infants, functional connectivity between the lesional (*grey*) and non-lesional hemisphere (*white*) is largely preserved even in the presence of focal brain pathology. The exception is case 1 at 1 year of age, who has lost inter-hemispheric connectivity between the perirolandic regions and has functionally ‘disconnected’ ipsilesional basal ganglia. Intra-hemispheric connectivity between the perirolandic regions and the supplementary motor area (SMA) is absent in the lesional hemisphere in all cases at term equivalent age and the majority at 1 year of age. Node sizes are scaled by degree (the number of connected edges), node colour is scaled by betweeness centrality (a measure of the amount of control that a node exerts over the interactions of other nodes in the network), and edge thickness is scaled by the pairwise Pearson’s partial correlation coefficient. Only edges which survived a false discovery rate correction (FDR) correction of *p* < 0.05 are shown

Functional connectivity analysis revealed significant inter-hemispheric connectivity between the left and right insular/opercular and perirolandic regions in all control infants, with additional bilateral connectivity to the SMA (Fig. 4). Consistent functional connectivity was also seen between the thalami and adjacent basal ganglia and bilaterally between the two thalami. Similarly in case infants, inter-hemispheric functional connectivity was largely preserved between the perirolandic and insular/opercular regions at both time-points. At TEA, connectivity was not seen between the SMA and perirolandic area in the lesioned hemisphere, in contrast to the non-lesioned side. The exception was case 1, who had marked sensori-motor network disruption at both time-points, including a loss of functional connectivity between the perirolandic regions and complete functional ‘disconnection’ of the ipsilesional basal ganglia at 1 year.

## Discussion

In infants who have suffered unilateral HPI and developed unilateral spastic cerebral palsy, alterations in the emerging architecture of sensori-motor functional and structural connectivity can be characterized in early infancy using advanced MRI techniques. We describe the normal framework of connectivity at TEA and provide evidence which suggests that differences due to perinatal brain injury are already present at TEA and precede the manifestation of later neurological and neurodevelopmental deficits. Regardless of focal brain pathology, functional responses were consistently seen in the perirolandic region contralateral to the side of passive sensori-motor stimulation, with the thalamo-cortical tracts appearing to have developed compensatory trajectories which circumvented areas of damage to reach the identified functional activity. In contrast, marked asymmetry in the volume and microstructure of the efferent CSTs was seen at TEA in the presence of HPI. Functional connectivity analysis suggests that homotopic inter-hemispheric connectivity is integral to the sensori-motor network in early infancy and is largely preserved even following focal brain injury.

Our findings are in agreement with studies of older children and adolescents with similar patterns of periventricular brain injury, in which contralateral somatosensory functional responses to passive sensori-motor stimulation were also consistently seen [23, 24]. In these cases, reorganization of somatosensory responses to the ipsilateral cortex appears to be rare and is associated with abnormal sensory function [24]. While we saw bilateral functional responses in two of the cases at TEA (and one of the controls), they are also observed in 40–60 % of healthy infants and are thought to occur through inter-hemispheric (transcallosal) connections rather than a direct ipsilateral pathway [9]. Although an altered distribution of functional responses following perinatal brain injury has been described in case reports, this was not seen in our study [7, 8]. Of importance, the perirolandic region was structurally intact in all of our cases, and therefore, our findings support the hypothesis that the location and functional role of the primary somatosensory cortex are determined before or very early in the third trimester [23]. At this juncture, thalamo-cortical connectivity has not been fully established as the afferent axons are still growing through the subplate to reach their cortical targets, thereby allowing the establishment of the identified compensatory tract trajectories seen in infants with unilateral HPI at TEA [1].

Unilateral perinatal brain injury has been previously shown with diffusion MRI to result in marked impairment of the volume and microstructure of the CST in the lesioned hemisphere at TEA [18]. In the early third trimester, the efferent CSTs are fully established and therefore cannot develop compensatory trajectories following injury acquired during this period, in marked contrast to the growing afferent thalamo-cortical tracts [8, 23, 25]. Asymmetry in CST volume in the neonatal period was sustained at 1 year of corrected age, which may represent impaired development of the ipsilesional fibres or possible hypertrophy of the contralesional fibres (or a combination of both) [8, 25]. While asymmetry of the CST was also observed in markers of microstructural integrity (FA and RD) at TEA, we did not find this at 1-year corrected age despite a clear functional manifestation (haemiparesis). This discrepancy may represent limitations inherent to the diffusion tensor model which struggles to model white matter microstructure in areas with inherently low anisotropy such as those containing crossing fibres [26]. Furthermore, as it was not possible to assess the functional activity of the primary motor cortex in these neonatal subjects, the tractography analysis of the CSTs could not be guided by the identified functional activity in the same way as the thalamo-cortical pathways. This would have been of great interest, as haemiparesis is often associated with consolidation of the ipsilateral CST from the healthy hemisphere to the paralyzed limb [8, 25]. Further studies of both the afferent and efferent pathways could therefore benefit greatly from additional data derived through other functional imaging techniques (such as magneto-encephalography (MEG), electro-encephalography (EEG), or transcranial magnetic stimulation (TMS)) which can offer complementary information about pathway function with greater temporal specificity.

Sensori-motor network analysis revealed intact inter-hemispheric functional connectivity between the perirolandic regions in early infancy even in the presence of large brain lesions. This relationship was impaired only in case 1 at 1 year, which was notable given their poor neurodevelopmental outcome. In contrast, bilateral functional sensori-motor responses and preserved inter-hemispheric perirolandic connectivity were seen in case 3 at both time-points, who had the best neurodevelopmental outcome of the cases. Taken together, these results suggest that in the presence of a focal pathology and impaired CST development, preserved inter-hemispheric functional connectivity may be vital for positive neurodevelopmental outcome. Furthermore, this finding is in keeping with those of animal studies of hypoxic injury and in children following traumatic brain injury, which also observed a significant relationship between transcallosal connectivity and later neurobehavioural outcome [27, 28]. Of additional interest, we also found that intra-hemispheric functional connectivity between the perirolandic cortices and the SMA was impaired at TEA in the lesional hemisphere. The SMA is functionally active even during early infancy, and functional responses are consistently elicited with sensori-motor tasks [9, 15]. It has an important role in adult post-stroke recovery, with acute-phase ‘hyper’-connectivity correlating with good motor recovery and increased activity seen during voluntary tasks chronically [4, 5].

Our work is limited by the small sample size and restricted time-points of assessment, and further longitudinal work will therefore be vital to build on our findings. These limitations are also pertinent given that all diffusion tractrography algorithms are susceptible to the identification of false positive and negative tracts (particularly at a single subject level) and given that MR-derived markers of white matter microstructural integrity are known to change during normal human development in addition to following injury [26]. To provide a precise characterization of the specific effects of a focal brain lesion, here, we intentionally studied only infants with similar injury in terms of location and timing, although further work is needed to study the effects of different perinatal lesions as they have been found to result in marked differences in neuroanatomy and functional outcome in later childhood [8, 23]. Given our specific study group, the effects of HPI on functional connectivity were explored only in the sensori-motor network in the context of their later cerebral palsy, although it would certainly be of interest to study the wider effects on other functional networks. We were also unable to fully explore some of the key relationships in the network due to constraints imposed by EPI acquisition sequences, which made it necessary to include more than one area (such as M1 and S1) in single masks and exclude other important structures (the cerebellum) entirely from our analysis. Future work would therefore also benefit greatly from acquiring diffusion-weighted data which would allow an improved estimation of white matter fibre orientation over the limited diffusion tensor model used here, such as what can be achieved with high-angular-resolution diffusion-weighted imaging (HARDI) [26]. Finally, in order to fully elucidate the functional role of the identified afferent and efferent pathways, it will also be necessary to collect higher temporal resolution data about the amplitude and morphology of the electrophysiological evoked responses within the pathological hemisphere using techniques such as EEG or MEG.

## Conclusions

In summary, in this study, we demonstrate the feasibility of applying fMRI and diffusion tractography to offer new insights into the early effects of acquired perinatal brain injury in a small case series of infants with a clearly defined pattern of injury. We found that at TEA, functional responses to passive sensori-motor stimulation are in the contralateral perirolandic cortex, with established thalamo-cortical and symmetrical CST structural connectivity and a consistent pattern of inter-hemispheric functional connectivity. In the presence of unilateral HPI, passive sensori-motor functional responses remain in the lesional hemisphere, with an apparently altered but intact pattern of thalamo-cortical structural connectivity. In contrast, unilateral deficit of CST integrity at TEA is predictive of later contralateral motor impairment, and altered inter-hemispheric functional connectivity is associated with poor neurodevelopmental outcome. This small data series suggests that MRI in early infancy has a marked potential to provide new information regarding the pathophysiology of cerebral palsy, could be used as a possible biomarker for testing future therapeutic interventions and may provide new clinical prognostic information.

## Electronic supplementary material

Below is the link to the electronic supplementary material.

ESM 1 (GIF 64 kb)

High Resolution Image (TIFF 9482 kb)
