# Supplementary figures and images for: The effects of hemorrhagic parenchymal infarction on the establishment of sensori-motor structural and functional connectivity in early infancy
Source: Neuroradiology. 2014 Aug 14;56(11):985–94. doi: 10.1007/s00234-014-1412-5 (PMC4210651; doi:10.1007/s00234-014-1412-5)

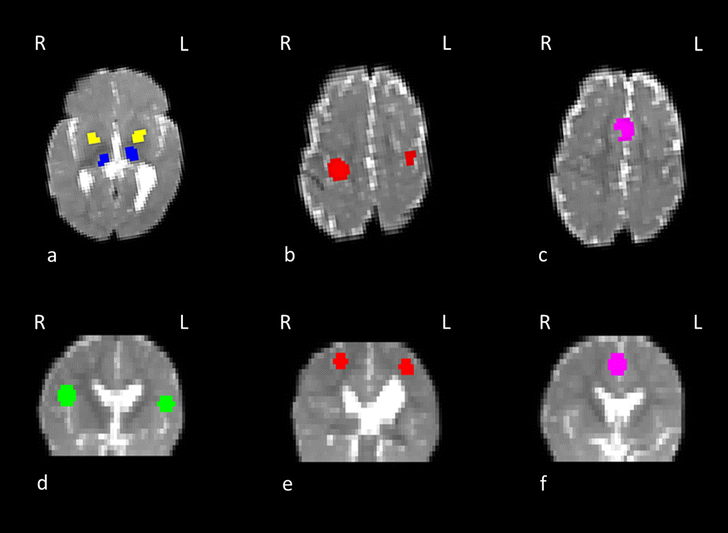

Supplement: Supplementary file 1 — (GIF 64 kb) [file 234_2014_1412_Fig5_ESM.gif]

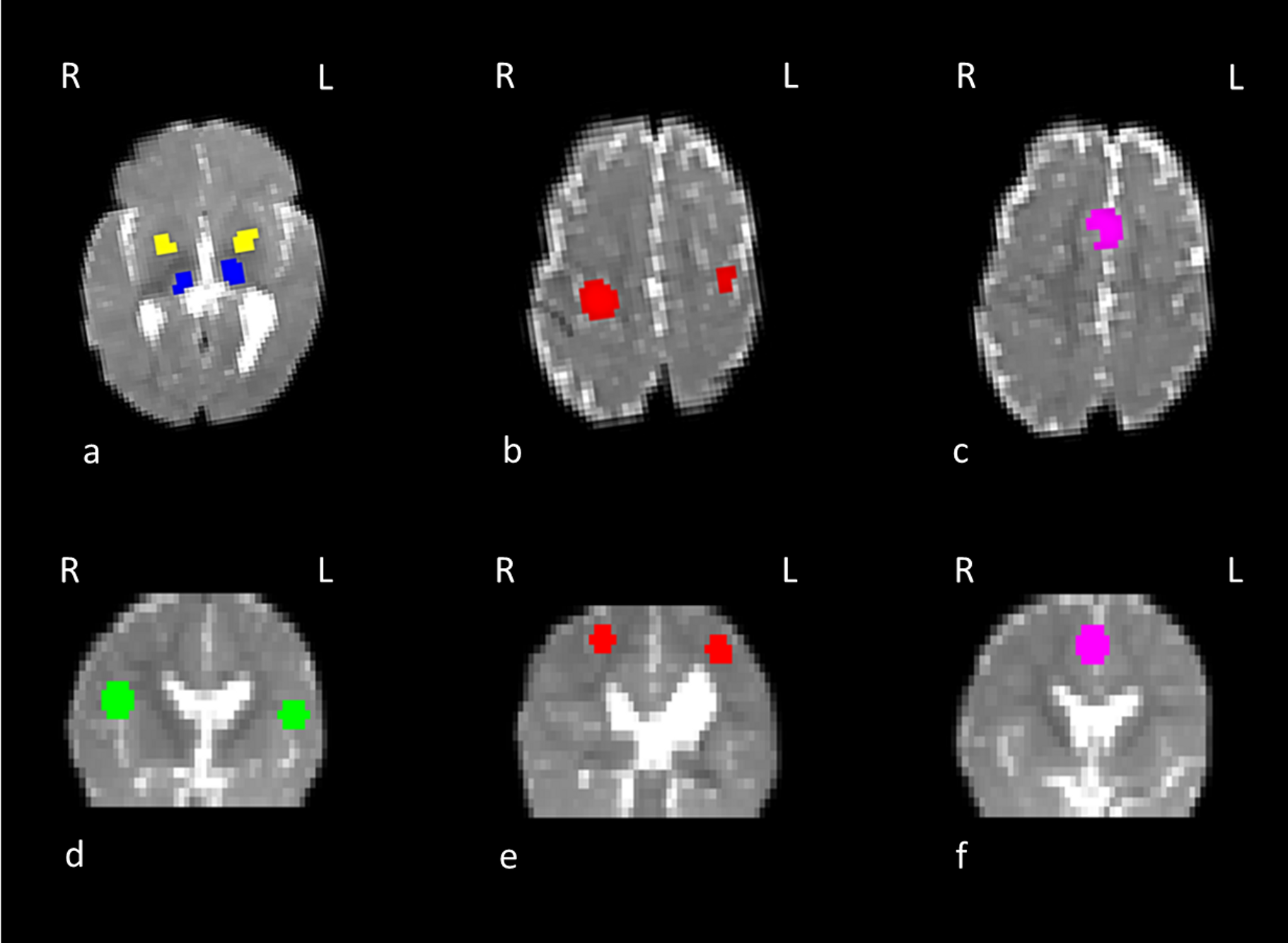

Supplement: Supplementary file 2 — High Resolution Image (TIFF 9482 kb) [file 234_2014_1412_MOESM1_ESM.tiff]
